# ^18^F-fluorodeoxyglucose positron emission tomography combined with computed tomography for bladder cancer staging: Diagnostic accuracy and prognostic implications

**DOI:** 10.14440/bladder.0044

**Published:** 2025-10-06

**Authors:** Tran Ngoc An Huynh, Xinyi Wei, Samiha Arulshankar, Darren Lam, Kylie Yen-Yi Lim, James Huang, Nieroshan Rajarubendra, Kevin Chu, Scott Donnellan, Weranja Ranasinghe

**Affiliations:** 1 Department of Urology, Monash Health, Melbourne 3168, Victoria, Australia; 2 Faculty of Medicine, Nursing and Health Science, Monash University, Melbourne 3800, Victoria, Australia; 3 School of Clinical Sciences, Monash University, Melbourne 3800, Victoria, Australia

**Keywords:** Bladder cancer, Computed tomography, ^18^F-fluorodeoxyglucose positron emission tomography combined with computed tomography, Lymph node staging, Overall survival

## Abstract

**Background:**

Muscle-invasive bladder cancer has a poor prognosis. Accurate lymph node (LN) staging is crucial, yet conventional imaging demonstrates limited sensitivity for detecting metastasis, necessitating improved pre-operative assessment.

**Objectives:**

To evaluate the diagnostic accuracy of ^18^F-fluorodeoxyglucose positron emission tomography combined with computed tomography (FDG-PET/CT) compared to computed tomography (CT) for LN staging in patients with bladder cancer(BC) undergoing radical cystectomy (RC), and to assess the impact of imaging-determined nodal status on survival outcomes.

**Methods:**

This retrospective study analyzed 138 patients who underwent RC and pelvic LN dissection (PLND) at a multisite tertiary institution from 2008 to 2021. All patients received either pre-operative CT or FDG-PET/CT within 8 weeks before RC and PLND. Sensitivity, specificity, positive predictive value (PPV), and negative predictive value for detecting LN metastasis (LNM) were calculated using pathological analysis as the reference standard. Kaplan–Meier survival analysis and Cox regression were employed to assess overall survival (OS).

**Results:**

FDG-PET/CT exhibited higher sensitivity (70% *vs*. 23.3%) and PPV (70% *vs*. 38.46%) compared to CT, but lower specificity (78.57% *vs*. 85.7%). Survival outcomes showed statistically significant differences in OS between node-positive and node-negative groups in the FDG-PET/CT cohort, but not in the CT cohort.

**Conclusion:**

FDG-PET/CT provides superior sensitivity and PPV compared to CT for LN staging in BC, facilitating more accurate pre-operative evaluation. The improved prognostic stratification associated with FDG-PET/CT may guide individualized treatment strategies.

## 1. Introduction

Bladder cancer (BC) represents the 10^th^ most common cancer across the globe, with approximately one-quarter of cases presenting as muscle-invasive BC (MIBC). MIBC is an aggressive form of BC that carries a high risk of metastasis and poor outcomes.^1^ The 5-year survival rate for MIBC remains below 50% even with radical surgery, underscoring the urgent need for effective pre-operative staging and treatment strategies to improve prognosis.^2^

Accurate lymph node (LN) staging is essential for determining optimal treatment, as LN metastasis (LNM) is associated with significantly poorer outcomes. About 25% of patients with stage T2 MIBC and up to 50% with stage T3 MIBC present with LNM.^3^ The presence of LNM may significantly alter treatment decisions, impacting the extent of surgical resection, the use of adjuvant therapies, and overall prognosis. Patients with accurate staging are more likely to receive tailored interventions, potentially improving survival rates and quality of life. Conventional imaging modalities, such as computed tomography (CT) and magnetic resonance imaging, are often used to assess LN status by measuring LN size, with pathologically enlarged nodes generally defined as those exceeding 8 mm in the pelvic region or 10 mm in the abdomen along the short axis.^4^ However, LNM in BC may cause minimal LN enlargement, resulting in limited sensitivity. In addition, benign conditions can lead to LN enlargement, which further reduces specificity. Consequently, CT’s accuracy in detecting LNM ranges from 73% to 92%.^5^

An emerging alternative is ^18^F-fluorodeoxyglucose positron emission tomography in combination with CT (FDG-PET/CT), which provides metabolic imaging that may enhance the detection of LNM compared to CT alone.^1^ FDG-PET/CT offers several potential advantages over conventional imaging by identifying metabolically active cancer cells, even in LNs that are not significantly enlarged. This can lead to earlier detection of metastatic disease, potentially improving treatment planning and patient outcomes. By combining functional and anatomical information, FDG-PET/CT may provide a more comprehensive assessment of tumor burden and show promise in improving the pre-operative staging of LN status in MIBC patients, potentially guiding more tailored treatment decisions.

This study aimed to compare the sensitivity and specificity of FDG-PET/CT with CT alone for assessing LN status in BC patients who had undergone cystectomy at a multisite tertiary center. The secondary aim included evaluation of the survival outcomes between patients with clinical node-positive and node-negative BC on FDG-PET/CT and CT alone.

## 2. Materials and methods

This retrospective study reviewed all patients who had undergone radical cystectomy (RC) for BC at a multisite tertiary institution between January 1, 2008, and June 30, 2021. Cases were identified through Medicare Benefits Schedule codes specific to RC operations. Patients were included if they underwent a pre-operative imaging assessment using either CT or FDG-PET/CT within 8 weeks before RC, and underwent a pelvic LN dissection (PLND) intraoperatively. Patients were excluded if they received neoadjuvant chemotherapy (NAC). All cases were reviewed at the departmental multidisciplinary tumor board (MDT) meeting before surgery to establish individualized treatment plans. The histopathology of the transurethral resection of bladder tumor specimen was reviewed by a uropathologist, and pre-operative images were assessed by a dedicated uroradiologist during the MDT discussion. This study was approved by the local institutional research ethics board (Ethic Committee Name: Monash Health Human Research Ethics Committee; Approval Code: RES-23-0000-581Q).

### 2.1. Imaging techniques

Patients underwent pre-operative imaging as recommended by MDT, with either CT and/or FDG-PET/CT based on clinical indications. CT scans were performed with intravenous contrast according to standard protocols. LN positivity was determined by nodal size, with positive nodes defined as those measuring > 8 mm in the short axis for pelvic nodes, or > 10 mm for abdominal nodes. FDG-PET/CT scans were performed according to standard protocols from the skull base to the mid-thighs. To minimize ^18^F-fluorodeoxyglucose (^18^F-FDG) concentration in the urinary tract, a diuretic was administered concurrently with the injection of ^18^F-FDG. Imaging was conducted 120 min after radiopharmaceutical administration. LN positivity on FDG-PET/CT was defined by a maximum standardized uptake value (SUV_max_) threshold of > 2, with metabolically active nodes above this threshold considered positive for metastatic involvement.

The decision to proceed to FDG-PET/CT rather than CT alone was made at the MDT. FDG-PET/CT was preferentially ordered for several reasons, including equivocal nodal findings on CT and radiological or pathological high-risk features.

### 2.2. Surgical procedure

The indications for RC were either MIBC or high-risk non-muscle-invasive disease. RC and PLND were performed on all included patients. The extent of PLND was determined by the operating surgeon, based on pre-operative imaging findings, intraoperative factors, and MDT recommendations. Pathological analysis of dissected nodes was used to confirm LNM.

### 2.3. Data collection and outcomes

Primary outcomes were the sensitivity and specificity of FDG-PET/CT and CT in detecting LNM, using pathological analysis as the reference standard. Secondary outcomes included overall survival (OS) based on imaging-determined nodal status.

### 2.4. Statistical analysis

Descriptive statistics were used to summarize baseline demographic, clinical, and imaging characteristics. Sensitivity, specificity, positive predictive value (PPV), and negative predictive value (NPV) were calculated for each imaging modality. OS was analyzed using Kaplan–Meier survival curves and compared between node-positive and node-negative groups for each imaging modality using the log-rank and generalized Wilcoxon test. A multivariable Cox regression was utilized to adjust for potential confounders. Statistical analyses were performed by employing GraphPad Prism (v10, Dotmatics, United Kingdom), Statistical Package for Social Sciences (SPSS 29.0, IBM, United States), and R software (v4.4.1, R Foundation for Statistical Computing, Austria). *p*<0.05 was considered statistically significant.

## 3. Results

### 3.1. Patient characteristics

A total of 199 patients underwent RC at our tertiary health service between January 1, 2008, and June 30, 2021. Of these, 138 patients met the inclusion criteria of having either a CT and/or FDG-PET/CT within 8 weeks of RC with PLND. Among them, 114 underwent CT alone, and 24 received both CT and FDG-PET/CT.

Table 1 presents the demographic data and histological findings of the cohort. The median age of the patients was 74 years (range: 33–95). The sex distribution was 125 (80.65%) men and 30 (19.35%) women.

**Table 1:** Patient characteristics and histological findings on cystectomy specimen

Demographics	CT (*n*=138)	PET (*n*=24)	*p*-value
Age (years), mean (range)	71.4 (33–93)	64.2 (33–84)	0.006
Sex, *n* (%)			0.123
Male	112 (81.16)	22 (91.67)	
Female	26 (18.84)	2 (8.33)	
Interval between imaging and radical cystectomy, *n* (%)			
≤14 days	19 (16.23)	2 (8.33)	
>14 days	98 (83.84)	22 (91.67)	
Pre-operative cT stage, *n* (%)			0.860
cTa/T1	31 (23.85)	5 (20.83)	
≥cT2	99 (76.15)	19 (79.17)	
pT stage, *n* (%)			0.055
pT0	14 (10.14)	0 (0.00)	
pTis/Ta/1	35 (24.36)	11 (45.83)	
≥pT2	89 (64.49)	13 (54.17)	
Lymph node count, median (range)	10 (1–35)	11 (2–35)	0.128
pN stage, *n* (%)			0.844
pN0	96 (69.57)	16 (66.67)	
pN1	23 (16.67)	5 (20.83)	
pN2	16 (11.59)	2 (8.33)	
pN3	3 (2.17)	1 (4.17)	
Extent of lymph node dissection			0.058
Limited	8 (7.02)	2 (8.70)	
Standard	89 (78.07)	11 (47.83)	
Extended	16 (14.04)	9 (39.13)	
Super-extended	1 (0.88)	1 (4.35)	
Cystectomy histology			
Urothelial carcinoma, *n* (%)	117 (84.78)	18 (75.00)	0.314
Presence of concomitant CIS, *n* (%)	53 (38.41)	13 (54.17)	0.151
Pure squamous/adenocarcinoma, *n* (%)	8 (5.80)	3 (12.50)	0.359
Micropapillary, *n* (%)	4 (2.90)	1 (4.17)	0.388
Glandular, *n* (%)	4 (2.90)	1 (4.17)	0.388
Sarcomatoid, *n* (%)	1 (0.72)	0 (0.00)	0.319
Nested, *n* (%)	1 (0.72)	1 (4.17)	0.424
Other variants, *n* (%)	3 (2.17)	1 (4.17)	0.325

Abbreviations: CIS: Carcinoma *in situ*; CT: Computed tomography; cT: Clinical tumor stage; PET: Positron emission tomography; pN: Pathological lymph node stage; pT: Pathological tumor stage.

### 3.2. LN dissection

Among the 138 patients who underwent RC and PLND, the extent of PLND was quite consistent with most patients at our institution undergoing standard node dissections, with an overall median node count of 10 (range: 1–35). However, the median node count for patients who were also undergoing positron emission tomography (PET) scan was slightly higher, being at 11 (range: 2–35), which suggests node surgery was tailored toward patients and correlated with imaging findings, albeit not significantly.

Limited PLND, involving only the obturator and external iliac LN, was performed in 8 patients (7.02%). Standard PLND, including internal iliac LNs, was performed in 89 patients (78.07%). Extended PLND, including common iliac LNs, was performed in 16 patients (14.04%). Only 1 (0.88%) patient received super-extended PLND, also including para-aortic and paracaval LNs.

Metastatic LN invasion (pN+) was found in 42 patients (30.43%). Of these, four patients had limited LND, 23 had standard, seven had extended LND, and one had super-extended PLND. In addition, among those with pN+, four were pT1, seven were pT2, 24 were pT3, and seven were pT4.

### 3.3. Computed tomography vs. ^18^F-FDG positron emission tomography

Computed tomography was interpreted as suspicious of LN invasion in 43 cases (27.74%). The mean number of positive LN on CT was 1.67 ± 0.9 per patient, with a mean diameter of 11.62 ± 3.15 cm. FDG-PET/CT was interpreted as suspicious of LN invasion in 11 cases (37.9%). The mean number of positive LN on FDG-PET/CT was 1.09 ±1.14 per patient, with a median SUV_max_ of 4.7.

^18^F-FDG PET plus CT showed higher sensitivity (70.00%, 95% confidence interval [CI]: 34.75–93.33%) compared to CT (23.30%, 95% CI: 11.76–38.63%) but lower specificity (78.57%, 95% CI: 49.20–95.34%) compared to CT (85.70%, 95% CI: 77.84–91.61%). FDG-PET/CT also demonstrated a higher PPV of 70.00% against CT’s 38.46%, whereas the NPV was similar at 78.57% for FDG-PET/CT and 74.42% for CT. The diagnostic performance of CT and FDG-PET/CT for LN staging is summarized in Table 2.

**Table 2:** Diagnostic performance of CT and FDG-PET/CT for LN staging

Parameters	CT	FDG-PET/CT	*p*
Sensitivity	23.00% (95% CI: 11.76–38.63%)	70.00% (95% CI: 34.75–93.33%)	0.0101
Specificity	85.70% (95% CI: 77.84–91.61%)	78.57% (95% CI: 49.20–95.34%)	0.2695
Positive predictive value	38.46% (95% CI: 23.55–55.91%)	70.00% (95% CI: 44.16–87.32%)	0.1601
Negative predictive value	74.42% (95% CI: 70.82–77.71%)	78.57% (95% CI: 57.78–87.32%)	0.9599
Accuracy	68.39% (95% CI: 60.70–75.19%)	75.00% (95% CI: 53.29–90.23%)	0.8375

Abbreviations: CI: Confidence interval; CT: Computed tomography; FDG-PET/CT: ^18F^-fluorodeoxyglucose positron emission tomography combined with computed tomography; LN: Lymph node.

### 3.4. Subgroup analysis

The diagnostic performance of FDG-PET/CT and CT for LN staging was further analyzed across various clinical and pathological subgroups, as described in Table 3. These subgroups included the interval between imaging and RC (≤14 days *vs*. >14 days), history of Bacillus Calmette–Guerin (BCG) therapy (yes *vs*. no), tumor stage (≤pT2 vs. >pT3), the presence of concomitant carcinoma *in situ* (CIS) in the RC specimen, and the presence of variant histology (*e*.*g*., glandular, squamous, small-cell neuroendocrine, micropapillary, plasmacytoid, sarcomatoid, and nested), pure squamous/adenocarcinoma, and extent of PLND. Overall, FDG-PET/CT demonstrated higher sensitivity compared to CT across most subgroups, whereas CT exhibited a higher specificity. Factors such as variant histology, a history of previous BCG therapy, and the presence of concomitant CIS were found to influence the performance of both CT and FDG-PET/CT.

**Table 3:** Diagnostic performance of FDG-PET/CT and CT across clinical and pathological subgroups

Parameters	CT sensitivity % (range)	CT specificity % (range)	FDG-PET/CT sensitivity % (range)	FDG-PET/CT specificity % (range)
Interval between imaging and radical cystectomy (≤14 days)	14.3% (2.6–51.3%)	80.0% (49.0–94.3%)	100.0% (2.5–100.0%)	100% (2.5–100.0%)
Interval between imaging and radical cystectomy (>14 days)	24.1% (12.2–42.1%)	83.1 (73.7–89.7%)	60.0% (26.2–87.8%)	75% (42.8–94.5%)
Tumor stage (≤pT2)	25.0% (8.9–53.2%)	84.8% (75.3–91.1%)	66.7% (20.8 –93.9%)	75.0% (46.8–91.1%)
Tumor stage (≤pT3)	22.6% (11.4–39.8%)	87.9% (72.7–95.2%)	62.5% (30.6 –86.3	75.0% (30.1–95.4%)
Presence of concomitant CIS	15.8% (5.5–37.6%)	88.9% (76.5–95.2%)	66.7% (22.3–95.7%)	85.7% (42.1–99.6%%)
Presence of variant histology	33.3% (9.7–70.0%)	100.0% (67.6–100.0%)	100.0% (0.0–79.3%)	50.0% (9.5–90.5%)
Pure squamous/adenocarcinoma	100.0% (20.7–100.0%)	100.0% (20.7–100.0%)	66.7% (20.8–93.9%)	100.0% (56.6–100.0%)

Abbreviations: CIS: Carcinoma *in situ*; CT: Computed tomography; FDG-PET/CT: ^18^F-fluorodeoxyglucose positron emission tomography combined with computed tomography; pT: Pathological tumor stage.

### 3.5. Survival outcomes

Patients with imaging-determined LN positivity had worse survival outcomes than those with node-negative findings. For FDG-PET/CT, the 5-year OS rate was 0% (95% CI: 0.0–22.8%) for node-positive patients and 58.0% (95% CI: 29.1–76.8%) for their node-negative counterparts. For CT, the 5-year OS rate was 57.8% (95% CI: 35.2–80.2%) for node-positive patients and 58.0% (95% CI: 47.5–68.6%) for node-negative ones.

**Figure 1:**
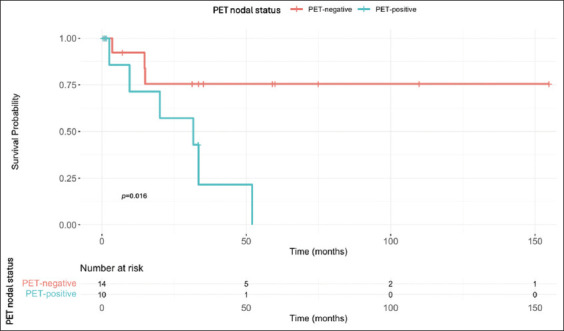
Overall survival curve for the positron emission tomography (PET) cohort

**Figure 2:**
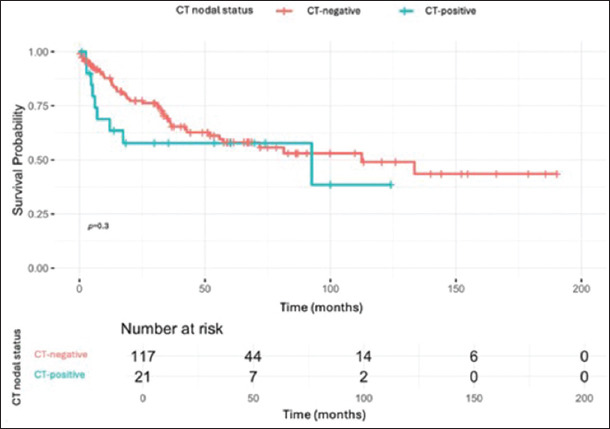
Overall survival curve for the computed tomography (CT) cohort

Kaplan–Meier survival curves are presented in Figures 1 and 2. In the FDG-PET/CT cohort, the difference in OS between clinically node-positive and node-negative patients was statistically significant based on both the log-rank and generalized Wilcoxon tests, with *p*-values of 0.0106 and 0.0312, respectively. However, in the CT cohort, no statistically significant difference in OS was observed, with a *p*-value of 0.2958 for the log-rank test and a *p*-value of 0.1936 for the generalized Wilcoxon test.

A multivariable Cox proportional-hazards model was constructed to identify independent predictors of OS (Table 4). After adjustment, pathological stage ≥T3 (hazard ratio [HR] 2.56, 95% CI: 1.34–4.89; *p*=0.004) and pure squamous or adenocarcinoma histology (HR 3.17, 95% CI: 1.12–8.97; *p*=0.030) remained independent predictors of poorer survival. In contrast, CT nodal status, variant histology, age, sex, CIS, perioperative chemotherapy (neoadjuvant or adjuvant), and an imaging-to-surgery interval >14 days were not significantly associated with OS.

**Table 4:** Multivariate Cox regression analysis

Variables	HR	95% CI	*p*
Stage ≥T3	2.56	1.34–4.89	0.004
Pure squamous/adenocarcinoma	3.17	1.12–8.97	0.030
Variant histology	0.29	0.07–1.29	0.104
Interval between imaging and radical cystectomy (>14 days)	0.84	0.33–2.13	0.714
CIS present	0.84	0.44–1.62	0.610
CT node	0.80	0.34–1.89	0.608
Neoadjuvant chemotherapy	1.17	0.44–3.13	0.753
Adjuvant chemotherapy	1.28	0.66–2.48	0.469
Age (year)	1.00	0.96–1.03	0.861
Male gender	0.87	0.41–1.86	0.716

Abbreviations: CI: Confidence interval; CIS: Carcinoma *in situ*; CT: Computed tomography; HR: Hazard ratio.

On univariable analysis, PET-positive nodal status was associated with a significantly increased risk of death (HR 4.80, 95% CI: 1.18–19.7; *p*=0.03). However, due to the small number of patients who underwent FDG-PET/CT (*n* = 24) and the fact that all PET-positive patients died, the variable exhibited quasi-complete separation. Therefore, the variable could not be included in the multivariable model.

## 4. Discussion

Accurate staging of BC is imperative for determining optimal patient management. The pathological stage of the primary bladder tumor, along with the presence of LNM, is a critical determinant of survival in patients with BC undergoing RC.^6^ Pre-operative staging of MIBC typically involves conventional staging with CT chest, abdomen, and pelvis. While CT is highly accurate in detecting primary BC, it has demonstrated a lack of sensitivity in nodal staging, given that it only detects enlarged LNs, subsequently leading to understaging of LN disease.^7^ To overcome this insensitivity in identifying LNM, FDG-PET/CT has been increasingly used in BC staging.

^18^F-FDG PET plus CT combines the functional capabilities of PET with the detailed anatomical information of CT, providing comprehensive information about both the metabolic and structural changes in the body. The use of ^18^F-FDG enables FDG-PET/CT to identify cells with high glucose uptake, such as neoplastic cells, allowing for early detection of locoregional disease and distant metastases before they become evident on conventional imaging, such as CT.^7^

In our study, the sensitivity of FDG-PET/CT in detecting LNM was significantly higher than that of CT across the entire cohort (70.0% *vs*. 23.0%, *p*=0.01) and most subgroups. Our findings align with a recent retrospective study by Al-Zubaidi *et al*.^8^ of 75 patients, which reported a sensitivity of 60.0% for FDG-PET/CT compared to 46.6% for CT. This underscores FDG-PET/CT’s ability to detect metabolically active nodes, enhancing its sensitivity, particularly for smaller or minimally enlarged nodes that may be missed by size-based criteria on CT.

The relatively low sensitivity of CT in our study (23.3%) is concerning and raises critical questions about the reliability of CT as a sole imaging modality for pre-operative LN staging in BC. This finding suggests that CT’s reliance on nodal size thresholds may lead to significant understaging of nodal disease, particularly in cases where LNM does not result in substantial LN enlargement. Consequently, patients with LNM may be incorrectly categorized as node-negative, potentially impacting treatment decisions and overall prognosis.

The specificity of FDG-PET/CT in detecting LNM in our study was comparable to that of CT (78.57% *vs*. 85.70%, *p*=0.27), aligning with findings from the study by Al-Zubaidi *et al.*,^8^ which reported a specificity of 83% for FDG-PET/CT and 100% for CT. The lower specificity of FDG-PET/CT indicates that false positives occurred, which may arise from inflammatory or reactive LNs exhibiting increased metabolic activity.^9^ This finding highlights the importance of confirmatory biopsy as an adjunct to FDG-PET/CT, particularly in cases where false positives may lead to overtreatment or unnecessary anxiety for patients.^8^

However, FDG-PET/CT demonstrated lower accuracy in staging pure squamous cell carcinoma and bladder adenocarcinoma, raising concerns about potential delays in NAC or RC without additional clinical benefits. Nonetheless, FDG-PET/CT showed higher sensitivity in variant histology and the presence of concomitant CIS. Although further research is required to validate these findings, this supports the potential role of PET-based imaging in guiding individualized treatment strategies in selecting patient populations.

In summary, FDG-PET/CT is advantageous over CT thanks to its higher sensitivity. This allows for better detection of metabolically active LNs and earlier identification of metastatic disease. This renders FDG-PET/CT a valuable tool in pre-operative staging of BC, particularly in patients with high-risk or ambiguous findings on CT. However, FDG-PET/CT’s lower specificity must be interpreted with caution, and where appropriate, with confirmatory diagnostic methods, such as biopsy.

Our survival analysis revealed a stark difference in OS for patients with PET-positive LNs. In the FDG-PET/CT cohort, the 5-year OS was 0% for node-positive patients compared to 58.0% for node-negative patients, whereas in the CT cohort, OS was similar for node-positive and node-negative patients (57.8% vs. 58.0%). This disparity likely reflects the dual influence of FDG-PET/CT’s ability to detect metabolically aggressive disease and potential selection bias, as FDG-PET/CT was more often performed in patients with high-risk features or equivocal CT findings. This suggests that FDG-PET/CT may serve not only as a diagnostic tool but also as a powerful prognostic indicator. Its ability to stratify patients based on tumor burden may enable more tailored treatment approaches, particularly in identifying those who may benefit from intensified multimodal therapy. These findings are in alignment with those of Mertens *et al.*,^10^ a retrospective study involving 211 patients, which demonstrated significantly shorter OS in patients with positive FDG-PET/CT findings compared to those with negative findings (median OS: 14 vs. 50 months, *p*=0.001). FDG-PET/CT was also identified as an independent predictor of mortality in that study. The ability of FDG-PET/CT to differentiate survival outcomes between node-positive and node-negative patients has significant clinical implications. By accurately identifying patients at higher risk of poor outcomes, clinicians can better balance the potential benefits and risks of RC. In certain cases, this may allow for the omission of RC when it is deemed futile, enabling a more tailored approach to patient management and resource allocation.^1^

In contrast, CT demonstrated limited prognostic value in our cohort. The 5-year OS rates for CT were 57.8% (95% CI: 35.2–80.2%) for node-positive patients and 58.0% (95% CI: 47.5–68.6%) for node-negative patients, with no statistically significant difference observed (*p*=0.2958 for the log-rank test). While CT remains a widely accessible and cost-effective imaging modality, its inability to reliably predict survival outcomes highlights its limitations in pre-operative prognostication for BC, particularly when compared to FDG-PET/CT.

Looking toward the future, integrating molecular and genetic evaluations alongside advanced imaging modalities holds promise for improving the risk stratification and management of BC. Transcriptome analysis has been shown to distinguish MIBC patients, and the analysis findings could further be correlated with imaging results to offer a more integrated approach to BC diagnosis and management.^11^ Future research should focus on combining these modalities and establishing more refined risk stratification models.

^18^F-FDG PET in combination with CT is also explored in prostate cancer, particularly for aggressive primaries and metastatic disease, though its sensitivity for nodal metastases is limited by low glycolytic activity. A study by Jadvar *et al.*,^12^ highlighted that FDG-PET/CT successfully detected 67% of LN and bone metastasis in patients. The parallels between FDG-PET/CT’s evolving role in prostate cancer and BC further underscore its potential to refine risk stratification, improve pre-operative staging accuracy, and guide more personalized treatment approaches in BC.

However, one of the primary limitations of FDG-PET/CT is the absence of a standardized SUV_max_ threshold for defining nodal positivity in BC. This is additionally a major limitation of the current BC literature, which contributes to the variability in sensitivity and specificity.^1^ The reported sensitivities for FDG-PET/CT ranged from 23% to 100%, while specificities ranged from 33% to 100%.^1^,^3^ Variability arose from differences in study methodologies, such as the use of visual analysis versus SUV_max_ thresholds, with inconsistent cutoff values. Factors influencing SUV_max_ include tumor biology, technical differences in PET scanners, and the reconstruction algorithms used for image processing.^3^ As a result, it is challenging to recommend a fixed SUV_max_ cutoff value for LN evaluation. In contrast, in prostate cancer staging, prostate-specific membrane antigen PET/CT cutoff values correlate well with cancer aggressiveness and are widely used in clinical practice.^13^ Hence, further research is needed to establish standardized criteria and protocols for interpreting FDG-PET/CT findings in BC staging.

Other limitations of FDG-PET/CT include its high cost, increased radiation exposure, and lack of an anatomical reference frame when CT is unavailable. Furthermore, FDG-PET/CT is not universally accessible, particularly in resource-limited settings, which may restrict its routine adoption into clinical practice.^7^

This study has several limitations. Its retrospective design and relatively small sample size for the FDG-PET/CT cohort may limit the statistical power to detect subgroup differences and introduce sampling bias. As a result, the findings should be interpreted cautiously. Larger, prospective multicenter studies are needed to validate these results, improve generalizability, and support the development of standardized FDG-PET/CT protocols for BC staging. Moreover, surgical protocols, including the extent of PLND, were not standardized across patients, which could influence pathological LN evaluation. This variability may affect the accuracy of pathological nodal staging, which serves as our reference standard for evaluating imaging performance. Limited dissections may miss metastatic nodes, resulting in false-negative pathology and underestimating the sensitivity of imaging modalities. Furthermore, we found that the median number of LNs removed was higher in FDG-PET/CT patients compared to CT (11 vs. 10) patients. While the difference was minor and not statistically significant, it might still influence pathological staging accuracy, which in turn could affect the calculated sensitivity and specificity of pre-operative imaging, potentially leading to further bias. In addition, all patients who underwent FDG-PET/CT also received CT. It is possible that FDG-PET/CT was performed selectively in cases where CT suggested suspected LNM or in patients with higher risk features. If so, this introduced a potential selection bias that needs to be acknowledged, as this may have impacted the observed sensitivity and specificity of FDG-PET/CT in our study. However, our study included only 24 patients who underwent both CT and FDG-PET/CT, making it important to compare the accuracy of both imaging modalities within this same cohort. The remainder of patients were only subjected to CT scans, limiting direct comparisons between the two modalities and therefore the generalizability and reliability of these findings.

## 5. Conclusion

This study highlights the superior sensitivity of FDG-PET/CT over CT in detecting LNM in patients undergoing RC for BC. FDG-PET/CT demonstrated significantly greater sensitivity, allowing for more accurate detection of metabolically active nodes that the size-based CT criteria may miss. While CT exhibited higher specificity, FDG-PET/CT’s ability to detect true nodal disease provides a more precise assessment of metastatic burden, reducing understaging.

Beyond staging, FDG-PET/CT emerged as a powerful prognostic tool, with imaging-determined nodal status strongly correlating with OS. Patients with PET-positive LNs had significantly worse survival outcomes, whereas CT failed to distinguish meaningful prognostic differences between node-positive and node-negative patients. This highlights the role of FDG-PET/CT in refining pre-operative risk stratification, ensuring that high-risk patients receive appropriately intensified therapeutic strategies.

## Data Availability

The dataset used and analyzed in the current study may be available from the corresponding author upon reasonable request and with approval from the relevant ethics committee.
